# Occupational Therapy Research in Schools: A Mapping Review

**DOI:** 10.1155/2020/5891978

**Published:** 2020-08-08

**Authors:** Patrícia L. de Oliveira Borba, Beatriz P. Pereira, Joana R. B. de Souza, Roseli E. Lopes

**Affiliations:** ^1^Department of Health, Education and Society, Federal University of São Paulo, Santos 11015-020, Brazil; ^2^Department of Occupational Therapy, Federal University of Paraíba, João Pessoa 58051-900, Brazil; ^3^Department of Occupational Therapy, Federal University of São Carlos, São Carlos 13565-905, Brazil

## Abstract

**Background:**

Throughout the world, schools have become an important place for professional integration for occupational therapists.

**Objective:**

To map the production of knowledge on research related to the keywords “occupational therapy” and “school.”

**Method:**

A mapping review was performed, searching the terms “occupational therapy” and “school” in the Scopus and Web of Science databases. The data were used to construct a descriptive map of the production of knowledge about occupational therapy and school. The following data were categorized and extracted: years of publication, journals of publication, authors' and coauthors' countries, descriptors, informant population, beneficiary population, place of research, and occupational therapy propositions.

**Results:**

It included 127 research articles covering from 1988 to 2017. This has been a scientific field under construction for at least 30 years, largely centralized in the United States of America, mostly dedicated to children, and focused on disabilities, with an emphasis on rehabilitation through descriptions and analyses of interventions for individuals or, when it was for groups, with the final goal of benefitting individuals with disabilities. *Implications*. Examining the existing scientific production invites us to reflect on whether the dominant focus in this field has responded to the contemporary problems of schools.

## 1. Introduction

The scientific field of a subject is defined by the production, dissemination, and discussion—both academic and professional—of knowledge [1]. In a globalized and connected world, it is necessary to understand the diversity of research on occupational therapy in general and, for the purposes of this study, what has been and is being produced on the subject/processes of occupational therapy in school.

In 2016, the World Federation of Occupational Therapy [2] published a document entitled “Position Statement on Occupational Therapy Services in School-Based Practice for Children and Youth,” stating the organization's position on the contribution of occupational therapy in promoting inclusive education for school-based practices for children and youths and also asserting that inclusive education is a paramount and nonnegotiable right. The WFOT's position [2] is in line with the concept of inclusive education postulated by the United Nations [3] which supports the rights of persons with disabilities in order to ensure that effective individualized support measures are provided in environments that maximize academic and social development.

Although the position of the World Federation was an important milestone for school-based occupational therapy (SBOT), we would like to propose a reflection based on the findings of Ainscow et al. [4], proposing the expansion of the possible contributions of occupational therapy to schools. Those authors developed a typology of six different ways of thinking about inclusion in education worldwide, stating that there is no one perspective on inclusion within a single country. Therefore, the WFOT's understanding of inclusion is just one of several possible concepts.

In addition, Ainscow et al. [4] problematized this way of defining school/educational inclusion as ineffective when it focuses solely on “disability” or “special education needs” not addressing the fact that any student's participation can be impaired or improved. Despite the conceptual choice of the WFOT, it is important to know whether their official perspective is supported by the field of scientific knowledge that occupational therapists worldwide have assembled. Would the population that is benefited, either directly or indirectly, from the proposals of occupational therapists' practices in schools be exclusively those who face educational barriers due to disability or other medical conditions? This was the central issue that motivated this study.

The school is an institution absolutely present in the lives of children, adolescents, and youths, which is a prominent place in the composition of these people's social and support networks. Because school is the place where young people are during a considerable portion of their everyday lives, it must be a priority in any interaction intended with them. The school also is a fundamental strategy in fostering and promoting projects that can guarantee access to a meaningful education in the 21st century. We share one of the greatest contemporary thinkers on education's concern regarding the demands that schools face in modern society:

“[...] we need to aim as much as possible at preparing students not only to be themselves, but also to enter society, but with the ability to be a culture producer in all fields, at least to have the ability to enjoy, to know enjoy all the contributions of civilization, the arts, the techniques, the literature. Culture must be directed to all, facilitating intellectual dispositions while forcing everyone with firm sweetness to learn and to participate in all human pleasures [5].”

Manacorda's studies, as a whole, problematize the school and its social function, allowing us to reflect on the role of occupational therapists within the institution of school and what they have been proposing in this area.

In this sense, it is important to understand the worldwide contributions of occupational therapists to school and whether they really address the demands of contemporary schools. This literature review contributes to that understanding by surveying and mapping the relevant publications in scientific journals and then identifies what has not yet been done [6].

The procedures followed to answer the research question are presented ahead.

## 2. Methods

A mapping review is aimed at providing an understanding of the scope of research activity in a given area [7]. This method was developed to map and categorize the existing literature on a particular theme and also to facilitate the identification of gaps in the research. It enables the description of a research field [8].

Systematic mapping is developed based on a coding strategy (the creation of reading keys), which is applied to obtain general variables (e.g., the country where each study was conducted and its target population) and complementary variables (often associated with the central theme considered in the article) [7].

In early 2018, a search was conducted in two databases: Scopus and Web of Science. Since the central research question was, “which populations are the knowledge produced in the interface of ‘occupational therapy and school' directed at?”, the keywords searched were “occupational therapy” and “school.” No filters were applied regarding year of publication or anything else, the aim being to find as many references as possible. The data was then used to construct a descriptive map of the available knowledge produced about occupational therapy and school. The year of the earliest text found was 1979, but the first research articles found were only published in 1988. Among the studies gathered, this review focused on texts categorized as research articles, which spanned the period from 1988 to 2017.

A total of 1521 texts (not just research publications) were found in the search, and after reading the titles, abstracts, and keywords, 190 texts addressing occupational therapy and school as a focus of occupational therapists were included in the review. The exclusion criteria were as follows: publications without abstracts; articles that did not include or address “occupational therapy” or its correlates (e.g., “occupational therapist” and “occupational-therapeutic”) in their abstracts, descriptors, or titles; manuscripts that did not include “school” or “education” in their abstracts, keywords, or titles; texts that used only the school as a place for collecting specific research data; articles that referred to school only to reach the target population (schoolchildren and adolescents) for participation in studies and research, but without addressing aspects of occupational therapy in that space; those that presented a school as a setting, context, or important place for the everyday lives of children, but without considering it as a space of assistance and action of occupational therapists; texts that used the term “school” to refer to training programs in occupational therapy or some other profession, addressing specific vocational training issues; and manuscripts that were not classified as articles in periodicals (e.g., book chapters, editorials, and notes). Publications found in both databases were considered only once.

The data were tabulated in a Microsoft Excel® spreadsheet and sorted into the following general categories: authors, author affiliation, country of origin of the authors, year of publication; journal, country of the periodical, and type of publication ((1) literature reviews; (2) theoretical essays, reflection articles, and other types of publication; (3) research article; and (4) case report).

A bibliometric analysis of the data in this general spreadsheet was performed using filtering, counting, and graphing tools, thus enabling an overview of the knowledge production on the theme in question. Bibliometrics is a technique used to measure the indexes of production and the dissemination of scientific knowledge, and its use is recommended in association with comprehensive analysis techniques (i.e., not purely quantitative) [9].

**Figure 1:**
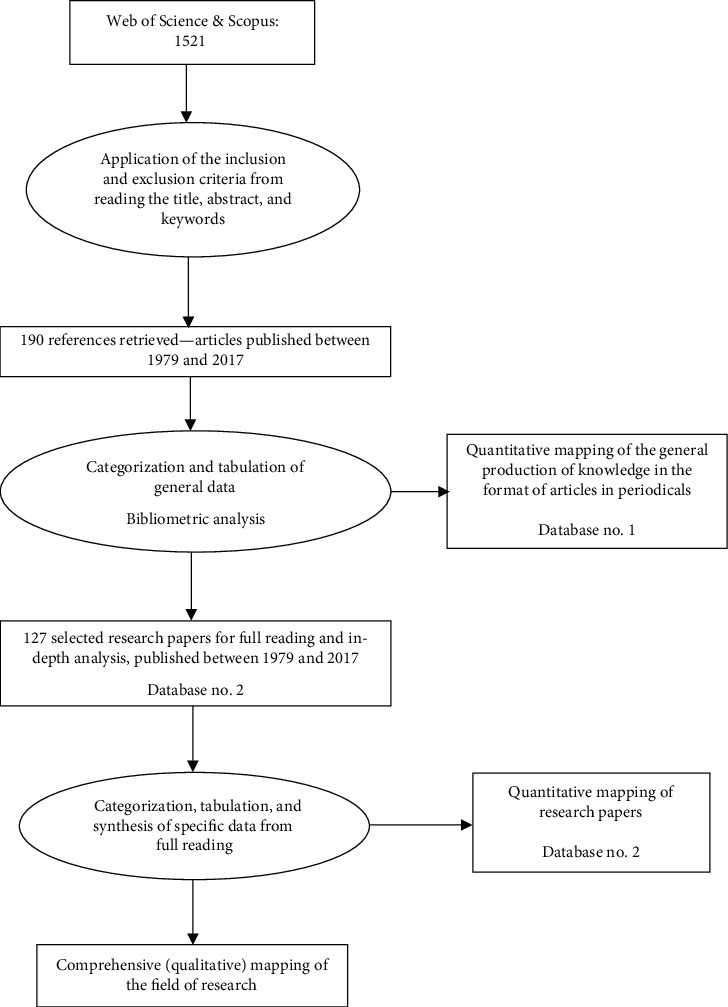
Flow of information through the different phases of the mapping review.

As research articles were the focus of this review, they were placed in a second spreadsheet and the following information about the texts was tabulated: informant population, target population, place of development, and main objective.  Figure 1 summarizes these steps and presents the quantitative statements for each of them.

Subsequently, the following section describes the 127 articles that composed the final selection, which are listed and numbered in the Supplementary Material. The different sets/groups that categorize these articles will be presented, and together with the quantitative data, comprehensive analysis will be provided to examine the research field on occupational therapy and school.

## 3. Results

### 3.1. Descriptive Analysis

**Figure 2:**
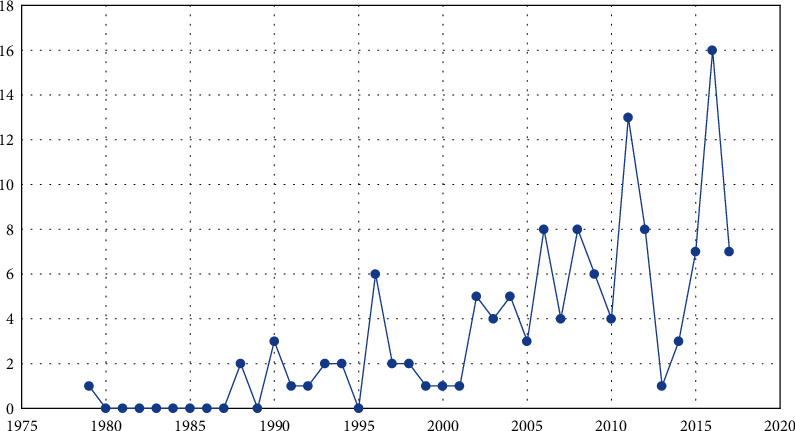
Number of research articles distributed according to the year of publication.

Figure 2 shows the number and time distribution of texts associated with research articles on the theme of occupational therapy and school published between 1979, the year of the earliest publication found, and 2017.

A certain irregularity was observed in the publication dates. Until 2001, the number of publications was usually very low. As of 2001, there has been an upward trend in the number of published studies, but there were important fluctuations during this period. The year with the smallest number of publications (three) was 2005, and the largest number came in 2011 (13), after which a significant reduction was observed through 2013, with only one published article. However, in 2014, this curve was once again increasing, reaching its peak in 2016, with 16 texts, a figure that dropped by over 56% in 2017, which saw seven publications. This may have been due to the fact that the American Occupational Therapy Association (AOTA) organized its 2014 annual conference with several lectures and communications that addressed the role of the occupational therapist at school [10].  Table 1 illustrates the number of texts selected per periodical.

Among the 28 different journals, the table shows a particular concentration of publications in the American Journal of Occupational Therapy (AJOT). Six other periodicals are highlighted, ranging from six to 16 texts, but the majority (18) of the journals published only one study each. In addition, it is important to notice that 64% (18 of 28) of the journals are from the United States of America (USA).

**Figure 3:**
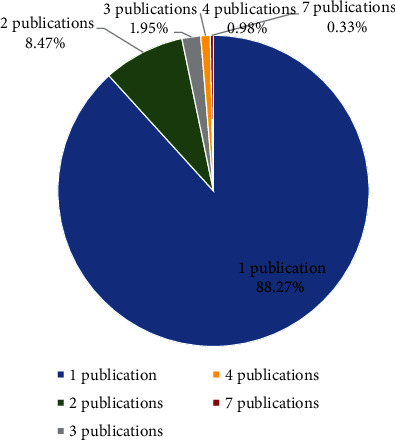
Frequency of publication in periodicals per authors and coauthors.

With respect to authorship and coauthorship, 305 names were gathered.  Figure 3 presents the frequency of publication in journals by authors and coauthors.

Authors and coauthors with only one published text comprise around about 88%. This demonstrates that this scientific field is still under construction, insofar as one-off experiments are prevalent, with no tradition of researchers studying the same object for years.

Regarding the first authors' countries of origin, is important to highlight the USA because it is home to 57.1% of the institutions with which the first authors were affiliated. It is followed by Canada (14.3%), Australia (12%), and Israel (4.7%); the other countries each had one to three institutions. Despite the incipient general nature of the field, it is noteworthy that Jane Case-Smith has seven published works between 1996 and 2014, which demonstrates her dedication to the theme.

By reading the abstracts of these 127 research articles, it was possible to identify their informant population and sort them by frequency: occupational therapists (42), children (37), teachers (21), adolescents (6), documents (5), parents/legal guardians (5), occupational therapy students (3), program managers/school principals (2), and others (6) (including occupational therapists' assistants (1), physiotherapists (1), speech-language pathologists (1), and professionals working with well-being (1), in classrooms (1), and with special education (1)). In this case, the sum is not equal to the total number of the research articles, because quite often, there was more than one informant population in a study.

Most of the studies addressed the confluence of occupational therapy and school by aiming at bringing direct “benefits” to children (98). Adolescents/youths were targeted in 14 studies; another 10 were focused on possible contributions to occupational therapists, that is, they turned to the discussion of the professional training necessary for them to work in the education sector.

Although each country systematizes and names levels of education in a particular way, we adopted the standard proposed by the Indicators of Education Systems of the Organization for Economic Co-operation and Development [11] to describe the data in this regard. Thus, research was developed in the following: primary education (91), early childhood education (15), secondary education (6), rehabilitation clinics/services (5), online (3), special education schools (2), schools of early childhood education and primary education simultaneously (2), rural schools (1), and schools of tertiary education (1).

The central/main themes of each research article were listed and analysed. These themes indicated possible propositions and perspectives regarding occupational therapy practice for and in schools or were related to the demands that each school presented to the occupational therapists. It is worth emphasizing that we have decided not to quantify these data because, although the themes were not the focus of the studies, they often dialogued with each other, and therefore, we have chosen to describe and discuss them in the following subitem.

### 3.2. Qualitative Analysis

In ordering the research articles on “occupational therapy” and “school,” a basic approach is to sort them based on what they express about professional practice. They are mostly dedicated to children with disabilities, specifically those aged between seven and twelve years.

“Disability,” despite being the majority theme that unites many of the research articles, encompasses situations of different complexities, differing from each other and requiring a variety of interventional responses. Another majority aspect is individualized assistance; although school is a collective and/or group context, most of the studies described and analysed interventions at the individual level.

This section presents the content analyses of these research publication sets, which are referred by their corresponding numbers, according to the Supplementary Material.

Of the studies included in the review for analysis, 10 texts (107, 124, 123, 91, 46, 45, 90, 88, and 105) addressed the problem of the inclusion of children with disabilities in school. These research articles presented propositions that point to occupational therapy as an important profession to cope with the issues associated with the access/impairments/limitations of children with disabilities in school spaces. A group of 15 studies (108, 21, 65, 7, 122, 73, 96, 24, 39, 71, 107, 2, 45, and 124) addressed the implementation, analysis, and assessment of models, projects, and/or programs of occupational therapy for and in schools, mostly focusing on children with disabilities, in addition to offering recommendations for improving services and identifying how these practices can occur. Two works (115 and 45) criticized the focus of occupational therapy intervention on individual cases. These works posited that the characteristics of the school environment directly influence the participation of children with disabilities in school activities, suggesting that occupational therapists should consider all the parts of the equation: the children, their environment, and the tasks they perform.

Also noteworthy is study number 124 of the Supplementary Material, which emphasized the discriminatory language that is used to refer to people with disabilities, specifically in the school context, and held that occupational therapy has the responsibility to eliminate occupational discrimination and promote occupational justice in places where discrimination occurs in any way by modifying social barriers, thus contributing to the construction of a more inclusive society.

Lack of fine motor coordination, directly associated with handwriting problems, emerged as an issue of concern; it was addressed by 26 (text numbers 83, 34, 4, 68, 38, 29, 41, 19, 57, 113, 26, 74, 127, 49, 106, 102, 67, 119, 84, 3, 60, 53, 25, 126, 114, and 95) of the research articles included in this review. This number certainly stems from the fact that most of these studies were conducted in the US, where “handwriting training” is the main common reason for referral to occupational therapy in schools [12].

Therefore, as pointed out by the authors of study number 25, many of these studies attempt to provide evidence of the effects that occupational therapy interventions in school have on students' handwriting. Thus, they address the implementation and evaluation of programs to promote the development of handwriting and writing fluency, such as the Write Start (26 and 29) and Letter School (68 and 84) programs. They also analyse the instructions offered by handwriting teachers (3) in the US Common Core State Standards Initiative for handwriting teaching (3 and 34).

Another significant group of 12 articles (47, 82, 62, 50, 54, 94, 80, 92, 70, 86, 109, and 101) provided examples of an established practice among occupational therapists in North America: “transition.” In the USA and Canada, this is a relevant field of professional activity, with many occupational therapists having been hired to form “transition teams” (teachers, parents, job coaches, vocational counsellors, and mentors); consequently, the studies describe their specificities and potentialities. The term “transition” refers to phases that imply some sort of change in the lives of persons with disabilities, and the occupational therapist is one of the professionals who can offer support for this change. Myers and Podvey [13] and Orentlicher [14] identified three transition phases in which occupational therapists have worked and the respective scientific productions: (1) transition of children with disabilities from early intervention services and/or rehabilitation to school (82, 92, 93, and 101), (2) transition of adolescents with disabilities from elementary school to high school (70), (3) transition of youths with disabilities to the labour market and/or adult life (47, 62, 54, 80, 86, and 109). These studies, in addition to describing the intervention work, evaluated the results of these services with the children/adolescents, parents, teachers, and managers, offering suggestions for increased effectiveness.

It is evident that the dominant theme of this field is children with disabilities. However, there are two other sets of themes that occupy space in this scientific field: mental health and social vulnerability. With respect to mental health, eight (13, 62, 125, 45, 87, 9, 6, and 28) studies are highlighted, among which three (9, 6, and 28) are large surveys carried out in the US to understand how the occupational therapists' intervention occurs in situations involving child psychosocial problems. Study number 107 specifically addressed child depression, and the authors concluded that sensory integration is the approach most frequently applied by occupational therapists as intervention response. They also criticized the training received by these professionals, claiming that it does not provide the necessary knowledge to inform their professional action regarding mental health needs. Two other studies addressed intervention for people on the autism spectrum: one focused on an analysis of the services provided to children with autism in the USA, identifying the lack of provision of some services, including occupational therapy (13), and the other assessed an online program for adolescents with autism, BOOST-A (62).

Regarding the studies included in the thematic group “social vulnerability situations” (five texts), two of them (104 and 97) analysed occupational therapy practice with children in situations of social vulnerability. The other three addressed youths in situations of social vulnerability in South Africa (40), Brazil (78), and Australia (36). The research conducted in South Africa examined the careers chosen by young refugees in a school in an urban periphery, pointing to gender issues as the main factors shaping unequal job perspectives between boys and girls. The Brazilian and Australian studies were both investigations involving occupational therapy interventions: the Brazilian study described, analysed, and evaluated the production of fanzines (community magazine) with poor youths in a school in the urban periphery where there is an illegal drug trade, whereas the Australian research focused on describing and assessing group interventions performed by occupational therapists with young refugees in two secondary schools, aimed at improving their participation in the school context. These works revealed interventions carried out from a collective perspective - which are exceptions in this field.

Another group of articles was directed at the professional practice of occupational therapy, describing, analysing, and evaluating services offered by occupational therapists, pointing out that there are two “models” of practice: direct assistance and collaborative consultation (43, 27, 72, and 23). Direct assistance would address the more individual needs of students who require specialized intervention strategies that can only be performed by occupational therapists, whereas collaborative consultation would address the problems faced by students by encouraging the entire school community to work together, and thus more effectively, on the educational goals planned for these students (43).

Studies dedicated to collaborative consultation comprised a total of 15 (111, 33, 81, 104, 23, 88, 89, 116, 115, 63, 15, 5, 27, 72, and 43) texts and reported how the collaboration between educators and occupational therapists can contribute to the effectiveness of proposals planned for the students. According to study number 14, in the USA, the results suggest that similar objectives can be achieved through direct assistance and collaborative consultation, but the relationship between occupational therapists and teachers leads to a more positive wider view related to the school and to the occupational therapy contribution in it.

Study 27, also in the USA, analysed the time that school occupational therapists spent using direct assistance or collaborative consultation to understand how this work had been conducted in relation to these models and to identify associated variables. These researchers identified advantages in the use of both models and seem to believe that children are better assisted when direct assistance is provided in combination with collaborative consultation, involving actions of both occupational therapists and teachers in the classroom.

In the same direction, study 107, in Israel, compared two models of rendering occupational therapy service in schools for children with intellectual disabilities: one group received direct assistance and the other received a combination of direct assistance and collaborative consultation, configuring what they called Collaborative Consultation for Participation of Students with Intellectual and Developmental Disability (Co-PID). The results showed a significant improvement in the participation of students in the group that underwent the Co-PID, whereas members of the group following the direct assistance model showed decreased participation.

According to studies 23, 88, and 89, all developed in Canada, occupational therapists who work in schools have recognized the need to move away from the direct assistance model and move towards a more collaborative approach in the classroom. In their opinion, Partnering for Change (P4C) is an innovative service model that has been contributing to improve collaborative consultation in schools. According to study 115, collaborative consultation has been widely adopted in occupational therapy practices in schools. The authors describe the collaborative work between teachers and occupational therapists in two cases. Ethnographic study methods were used to investigate multiple perspectives on collaborative school-based occupational therapy consultation for two students with disabilities in a region of Ontario, Canada. The intention was to contribute to the development of theoretical and empirical perspectives on the processes of collaborative work and the relation of these processes to the results achieved.

Along the same line, still in Canada, the authors of study 116 presented SBOT, which occurs within a complex system that includes the recipients of services, the service providers, and the decision-makers in programs in the health and education sectors. Despite the support of collaborative consultation at the political level, there would be, according to these authors, few practical guidelines available on how to coordinate interprofessional collaboration.

An expressive group of research articles (21) referred to themselves as “perception” studies, most of which already announce this in their titles, such as “Perceptions of Occupational Therapists regarding Service Delivery Models in School-Based Practice” or “Parent Perceptions of School-Based Occupational Therapy Services.” The texts are not dedicated to discussing the concept of perception. These studies were dedicated to analysing the perspectives and opinions of the parents of assisted children (82 and 10), teachers (11, 63, and 120), parents and teachers (30, 79, 32, and 49), teachers and managers (34), team partners (111) (physiotherapists and school coordinators), or the occupational therapist themselves (112, 77, 28, 121, 94, 18, 9, 123, 57, and 122).

Finally, there is a set of 10 research articles that focused on the training of occupational therapists to work in the field of education. Most of them included research based on questionnaires administered to occupational therapists or occupational therapy students (at different levels of training, from undergraduate students to doctoral candidates) and which discussed the “attitudes,” models/approaches, and knowledge necessary for the action of occupational therapists at school. The most recent student-focused survey conducted in four different countries, namely, Australia, the USA, the United Kingdom, and Taiwan (91), showed a favourable perspective regarding the proposal of inclusive education that legitimizes the work of occupational therapists in schools. On the other hand, studies focusing on occupational therapists (99) indicated the need to broaden and deepen knowledge to work in schools, and many pointed to continuing education and supervision to respond to the demands brought about by this activity (18), as well as to online training (75) and study groups (112).

## 4. Discussion

The analytical reading and systematization of the 127 articles revealed a range of themes and concerns that are present in the literature on “occupational therapy” and “school.”.

It is worth emphasizing the role of the USA in this field. In addition to having the largest number of professionals, researchers, journals, and institutions, apparently it was also the first country to regulate the work of occupational therapists at school. However, there is also no evidence of diversification in the production of knowledge, since an expressive set of research focuses on issues of motor rehabilitation, which we referred to as “handwriting,” and with a strong tendency to produce evidence on collaborative performance models of the occupational therapist at school, closely linked to physical rehabilitation.

It should also be remembered that the production of evidence in the US is closely intertwined with the needs of a capitalist and neoliberal country, where there is extensive privatization of the health services in which occupational therapists are usually included [15]. The data provided by the articles did not identify the position occupied by occupational therapists in supplying/rendering services in schools because most of the research articles were carried out by universities in partnership with schools and/or with health services together with schools. In any case, the need for occupational therapists to prove the effectiveness/efficiency of their work is directly associated with the context in which their service is “hired/rendered” in a market logic of “cost-benefit” for the consumer.

Polichino [16] suggested that occupational therapists are capable of making contributions to a wide range of issues. In fact, the first document published in the American Journal of Occupational Therapy (AJOT), an official publication of the American Occupational Therapy Association (AOTA), which provided the first guidelines for this field [17], suggested that occupational therapy in schools could address more than just “handwriting training.” In contrast, it is worth mentioning that legislative documents regulating professional practice in countries such as the US are more established [18, 19], which does not necessarily encourage diversification of the possibilities of practices.

It should be noted that the selected publications enabled understanding of the themes and possibilities in the scope of research regarding occupational therapy and school based on the assumption that, with the discussions of the models, approaches, theories, and techniques used, the role of occupational therapists is diversified and strengthened by the existing problems in the field of school education. However, one of the central results is that the research indicates that occupational therapists working on school inclusion processes tend to be mostly focused on children with disabilities, and they provide their services with a focus on one individual. Responding to our initial question, this fact mirrors the concept of inclusion in education proposed by the UN [3] and is in line with the perspective of the WFOT [2].

The WFOT [2] took an important step towards strengthening the field since it is the position of an organization that intends to speak with and on behalf of occupational therapists worldwide, legitimizing (or not) trends. In addition, the WFOT brought the discussion on social justice closer to the scope of occupational therapy in education. In order to build on this important step to continue constructing and strengthening this field, it is necessary to reflect on whether the WFOT [2], when speaking to occupational therapists around the world, actually welcomes other existing ways of looking at inclusion, occupational therapy, and school. Surely, it is up to us, who are interested in this field, to question whether such a concept is appropriate to each of our social, historical, and political contexts in order to guide the propositions of occupational therapy in our schools. Based on the mapping performed, it seems that a more welcoming position is necessary. In that direction, Ainscow et al. [4] proposed an expanded approach, addressing inclusion in education based on principles that are applicable in both education and society at large. Briefly,

“[...] inclusion is concerned with all children and young people in schools; it is focused on presence, participation and achievement; inclusion and exclusion are linked together such that inclusion involves the active combating of exclusion; and inclusion is seen as a never-ending process. Thus an inclusive school is one that is on the move, rather than one that has reached a perfect state. (p.25).”

Therefore, in addition to thinking about all the populations that may not be accessing or benefiting from school, this perspective demands more collective approaches in addition to individual assistance and action; in other words, if the focus of intervention by occupational therapists is kept mostly centred on the individual, this will hardly bring about the much desired inclusion. It demands the establishment of an inclusive common sense, which will naturally reflect also in school.

## 5. Conclusions

This review confirmed that indeed the school is an important focus for occupational therapists, and the relevant field of scientific knowledge focuses on children with disabilities as the population that is directly or indirectly benefited by the occupational therapists in schools. In addition, these actions have been conducted in an individualized way, despite the fact that school is and works in a collective context.

It is important to highlight that this study has clear limitations, since it provides reflections on research articles gathered through a search of only two databases which, although relevant to the academic environment, are restricted with regard to professional interests. Other databases have been left out of this survey, as well as other relevant productions that have been published in different forms, such as books.

There is a need for further research to address practices that broaden the target population and intervention approaches, potentiating and expanding the contributions of this area. For this to happen, it will be crucial that the training—both initial and continuing—of occupational therapists offers conditions for understanding the demands and needs present in society in relation to the school and the processes of schooling and that the school itself and the students are recognized as subjects who exist in a certain historical, political, and cultural period, so that it can move towards a democratic school based on radical inclusion, meaning that everyone can enjoy and be creative agents of culture and experience and promote social justice.

## Figures and Tables

**Table 1:** Number of studies published per journal.

Name of the journal	Country	Number of studies	%
*American Journal of Occupational Therapy*	USA	45	35.4%
*Australian Occupational Therapy Journal*	Australia	10	7.9%
*British Journal of Learning Disabilities*	United Kingdom	1	0.8%
*British Journal of Occupational Therapy*	United Kingdom	2	1.6%
*Canadian Journal of Occupational Therapy*	Canada	10	7.9%
*Cogent Education*	United Kingdom	1	0.8%
*Education 3-13: International Journal of Primary, Elementary and Early Years Education*	USA	1	0.8%
*Education and Training in Autism and Developmental Disabilities*	USA	1	0.8%
*International Journal of Adolescent Medicine and Health*	USA	1	0.8%
*International Journal of Disability Development and Education*	USA	1	0.8%
*International Journal of Inclusive Education*	USA	1	0.8%
*International Journal of Psychosocial Rehabilitation*	USA	1	0.8%
*Journal of Autism and Developmental Disorders*	USA	1	0.8%
*Journal of Occupational Therapy, Schools, and Early Intervention*	USA	16	12.6%
*Magallania*	Chile	1	0.8%
*Occupational Therapy in Health Care*	USA	2	1.6%
*Occupational Therapy in Metal Health*	USA	2	1.6%
*Occupational Therapy International*	United Kingdom	7	5.5%
*Occupational Therapy Journal of Research*	USA	6	4.8%
*Occupational Therapy Practice*	USA	1	0.8%
*OTJR Occupation, Participation and Health*	USA	1	0.8%
*Physical & Occupational Therapy in Pediatrics*	USA	9	7.1%
*Qualitative Report*	USA	1	0.8%
*Research Developmental Disabilities*	USA	1	0.8%
*Revista Brasileira de Educação Especial*	Brazil	1	0.8%
*Saúde e Sociedade*	Brazil	1	0.8%
*Scandinavian Journal of Occupational Therapy*	Denmark, Norway, Finland, Iceland and Sweden	1	0.8%
*Work: A Journal of Prevention, Assessment and Rehabilitation*	USA	1	0.8%
Total		127	100.0%

USA: United States of America.

## Supplementary Materials

Supplementary materials The supplementary material shows detailed dataset, its content is the list of articles that were included in the mapping review. Those marked with an asterisk are the ones that were cited on the text.
