# Supplementary material for: Occupational Therapy Research in Schools: A Mapping Review
Source: Occup Ther Int. 2020 Aug 8;2020:5891978. doi: 10.1155/2020/5891978 (PMC7455833; doi:10.1155/2020/5891978)
Supplement: Supplementary materials — The supplementary material shows detailed dataset, its content is the list of articles that were included in the mapping review. Those marked with an asterisk are the ones that were cited on the text. [file 5891978.f1.zip › 5891978.f1.pdf]

Supplementary Material: List of research articles.

	Title	Periodic	Year	Authors
1	Rater Reliability and Internal Scale and Person Response Validity of the School Assessment of Motor and Process Skills	*America Journal of Occupational Therapy*	1998	Atchinson, T. B., Fisher, A. G., Bryze, K.
2	*Item Test-Retest Reliability and Responsiveness of the School Outcomes Measure (SOM)	*Physical & Occupational Therapy in Pediatrics*	2008	Arnold, S. H., & Mcewen, I. R.
3	*Handwriting Instruction in Elementary Schools	*American Journal of Occupational Therapy*	2006	Asher, A. V.
4	*Handwriting Instruction in Elementary Schools: Revisited!	*Journal of Occupational Therapy, Schools, & Early Intervention*	2016	Asher, A., & Estes, J.
5	*Team Collaborative Practices Between Teachers and Occupational Therapists	*American Journal of Occupational Therapy*	2001	Barnes, K. J., & Turner, K. D.
6	*Perceptions Regarding School-Based Occupational Therapy for Children with Emotional Disturbances	*American Journal of Occupational Therapy*	2003	Barnes, K. J., Beck, A. J., Vogel, K. A., Grice, K. O., & Murphy, D.
7	*School-Based Occupational Therapy for Children with Fine Motor Difficulties: Evaluating functional outcomes and fidelity of services	*Physical & Occupational Therapy in Pediatrics*	2006	Bayona, C. L., Mcdougall, J., Tucker, M. A., Nichols, M., & Mandich, A.
8	Integrating Occupational Therapy Services in a Kindergarten Curriculum: A look at the outcomes	*American Journal of Occupational Therapy*	2009	Bazyk, S. S. et al.
9	*The Dilemma of Psychosocial Occupational Therapy in Public Schools	*Occupational Therapy in Mental Health*	2006	Beck, A. J., Barnes, K. J., Vogel, K. A., & Grice, K. O.
10	*Parent Perceptions of School-based Occupational Therapy Services	*Journal of Occupational Therapy, Schools, & Early Intervention*	2015	Benson, J. D., Elkin, K., Wechsler, J., & Byrd, L.
11	*Teachers' Perceptions of the Role of Occupational Therapist in Schools	*Journal of Occupational Therapy, Schools, & Early Intervention*	2016	Benson, J. D., Szucs, K. A., & Mejasic, J. J.
12	An Exploration of the Use of Occupational Language in School-based Occupational Therapy Documentation	*Journal of Occupational Therapy, Schools, & Early Intervention*	2015	Benson, J. D.
13	*Prevalence and Correlates of Educational Intervention Utilization Among Children with Autism Spectrum Disorder	*Journal of Autism and Developmental Disorders*	2016	Bilaver, L. A., Cushing, L. S., & Cutler, A. T.
14	The Sensory Integration and Praxis Tests: Illuminating struggles and strengths in participation at school	*OT Practice*	2006	Bodison, S. C., & Mailloux, Z. K.

15	*Reported Experiences from Occupational Therapists Interacting with Teachers in Inclusive Early Childhood Classrooms	*American Journal of Occupational Therapy*	2008	Bose, P., & Hinojosa, J.
16	Evaluation of the Effects of Sensory Integration-Based Intervention by a Preschool Special Education Teacher	*Education and Training in Autism and Developmental Disabilities*	2010	Bonggat, P. W., Hall, L. J.
17	Perceptions of Faculty Members in Special Education Concerning Occupational Therapy Services in Schools: A pilot study	*The Occupational Therapy Journal of Research*	1998	Bloom, D. T.
18	*School-Based Practice: Acquiring the knowledge and skills	*American Journal of Occupational Therapy*	2005	Brandenburger-Shasby, S.
19	*Validating the Use of the Evaluation Tool of Children's Handwriting-Manuscript to identify handwriting difficulties and detect change in school-age children	*American Journal of Occupational Therapy*	2012	Brossard-Racine, M., Mazer, B., Julien, M., & Majnemer, A.
20	Playful Interaction: Occupational therapy for all children on the school playground	*American Journal of Occupational Therapy*	2008	Bundy, A. C. et al.
21	*Contributions Made by Occupational Therapists in RtI: A pilot study	*Journal of Occupational Therapy, Schools, & Early Intervention*	2010	Cahill, S. M.
22	Using a Single-Subject Research Design to Evaluate the Effectiveness of Treatment	*American Journal of Occupational Therapy*	1998	Campbell, P. H.
23	*Support for Everyone: Experiences of occupational therapists delivering a new model of school-based service	*Canadian Journal of Occupational Therapy*	2012	Campbell, W. N., Missiuna, C. A., Rivard, L. M., & Pollock, N. A.
24	*Fine Motor Outcomes in Preschool Children Who Receive Occupational Therapy Services	*American Journal of Occupational Therapy*	1996	Case-Smith, J.
25	*Effectiveness of School-Based Occupational Therapy Intervention on Handwriting	*American Journal of Occupational Therapy*	2002	Case-Smith, J.
26	*Effectiveness of an Integrated Handwriting Program for First-Grade Students: A pilot study	*American Journal of Occupational Therapy*	2011	Case-Smith, J., Holland, T., & Bishop, B.
27	*Perceptions of Occupational Therapists regarding Service Delivery Models in School-Based Practice	*The Occupational Therapy Journal of Research*	1996	Case-Smith, J., & Cable, J.
28	*Variables Related to Successful School-Based Practice	*The Occupational Therapy Journal of Research*	1997	Case-Smith, J.
29	*Effects of a Classroom-Embedded Occupational Therapist–Teacher Handwriting Program for First-Grade Students	*American Journal of Occupational Therapy*	2014	Case-Smith, J., Weaver, L., & Holland, T.

30	*Parent and Teacher Perspectives in Collaborative Concepts of Therapeutic Programs for Students with ADHD	*Journal of Occupational Therapy, Schools, & Early Intervention*	2016	Chaimaha, N. & Chinchai, S.
31	Self-care at School: Perceptions of 6-year-old children	*American Journal of Occupational Therapy*	2005	Chaparo, C. J., & Hooper, E.
32	*The Perception of Occupational Therapy by Special School Teachers in Hong Kong.	*Occupational Therapy International*	1996	Chow, S. M., & Chung, J. C
33	*Promoting the Role of Occupational Therapy in School-based Collaboration: Outcome project	*Journal of Occupational Therapy, Schools, & Early Intervention*	2015	Christner, A.
34	*Handwriting and Common Core State Standards: Teacher, occupational therapist, and administrator perceptions from New York state public schools	*American Journal of Occupational Therapy*	2017	Collette, D., Anson, K., Halabi, N., Schlierman, A., & Suriner, A.
35	Barriers to the Use of Assistive Technology for Children with Multiple Disabilities.	*American Journal of Occupational Therapy*	2004	Copley, J. A., & Ziviani, J.
36	*Development and Evaluation of an Occupational Therapy Program for Refugee High School Students	*Australian Occupational Therapy Journal*	2011	Copley, J. A., Turpin, M., Gordon, S., & McLaren,
37	Handwriting Assessment of Franco-Quebec Primary School-age Students	*Canadian Journal of Occupational Therapy*	2016	Couture, M. et al.
38	*Practice Patterns of School-based Occupational Therapists Targeting Handwriting: A knowledge-to-practice gap	*Journal of Occupational Therapy, Schools, & Early Intervention*	2015	Cramm, H., & Egan, M.
39	*Occupational Therapy Practice in School Systems	*Physical & Occupational Therapy in Pediatrics*	1990	Crowe, T. K., & Kanny, E. M.
40	*Intercultural Partnering for the Benefit of South Africa Township High School Students	*Occupational Therapy International*	2015	Davis, J. Dodge, E., & Welderufael, M.
41	*Survey of Handwriting Instruction Practices of Elementary Teachers and Educational Programs: Implications for occupational therapy	*Occupational Therapy in Health Care*	2012	Donica, D. K., Larson, M. H., & Zinn, A. A.
42	Beyond Research Literature: Occupational therapists' perspectives on and uses of "evidence" in everyday practice	*Canadian Journal of Occupational Therapy*	2016	Dougherty, D. A., Toth-Cohen, S. E., & Tomlin, G. S.
43	*A Comparison of Service Provision Models in School-Based Occupational Therapy Services: A pilot study	*The Occupational Therapy Journal of Research*	1990	Dunn, W.
44	Survey of Interdisciplinary Activity Between Occupational Therapists and Speech Language Pathologists in The Public Schools	*The Occupational Therapy Journal of Research*	1992	Edwards, S., & Hanley, J.

45	*School Participation of Pupils with Physical and Psychosocial Limitations: A comparison	*British Journal of Occupational Therapy*	2009	Egilson, S., & Hemmingsson, H.
46	*Assistance to Pupils with Physical Disabilities in Regular Schools: Promoting inclusion or creating dependency?	*European Journal of Special Needs Education*	2009	Egilson, S. T., & Traustadottir, R.
47	*Characteristics of Students Receiving Occupational Therapy Services in Transition and Factors Related to Postsecondary Success	*American Journal of Occupational Therapy*	2017	Eismann, M. M., Weisshaar, R., Capretta, C., Cleary, D. S., Kirby, A. V., & Persch, A. C.
48	Handwriting Performance, Self-Reports, and Perceived Self-Efficacy Among Children with Dysgraphia	*American Journal of Occupational Therapy*	2009	Engel-Yeger, B., Nagauker-Yanuv, L., & Rosenblum, S.
49	*Teachers Perceptions of the Role and Effectiveness of Occupational Therapists in Schools	*Canadian Journal of Occupational Therapy,*	1993	Fairbairn, M., & Davidson, I.
50	*Career Counselling at School for Placement in Sheltered Workshops?	*British Journal of Learning Disabilities*	2012	Fasching, H.
51	Classroom-based Assessment: Validation for the school AMPS	*American Journal of Occupational Therapy*	2002	Fingerhut, P. et a.
52	Schoolwork Task Performance of Students at Risk of Delays	*Scandinavian Journal of Occupational Therapy*	2004	Fisher, A. G., & Duran, G. A.
53	*Assistive Technology and Handwriting Problems: What do occupational therapists recommend?	*Canadian Journal of Occupational Therapy*	2004	Freeman, A. R., Mackinnon, J. R., & Miller, L. T.
54	*A Qualitative Study of Occupational Therapy's Role in Adolescent Transition in a Midwestern Coalition of Many School Districts	*Journal of Occupational Therapy, Schools, & Early Intervention*	2011	Gangl, C., Neufeld, P. S., & Berg, C.
55	Consultoria Colaborativa em Terapia Ocupacional para Professores de Crianças Pré-escolares com Baixa Visão [Collaborative Consultation in Occupational Therapy for Teachers of Preschool Children with Low Vision]	*Revista Brasileira de Educação Especial*	2011	Gebrael, T., & Martinez, C. M.
56	Occupational Therapy Roles and Functions in the Education of the School-based Handicapped Student	*American Journal of Occupational Therapy*	1979	Gilfoyle, E. M. & Hays, C. W.
57	*Occupational Therapy Practitioners' Perceptions of Important Competencies for Handwriting Evaluation and Intervention in School-Aged Children	*Physical & Occupational Therapy in Pediatrics*	2011	Giroux, P., Woodall, W., Weber, M., & Bailey, J.
58	Facilitating Appropriate Referrals for Related Services in Schools	*Journal of Occupational Therapy, Schools, & Early Intervention*	2012	Goodrich, B., Hawkins, J., Burridge, A., & White, C.
59	Ethnographic Analysis: A study of classroom environments	*American Journal of Occupational Therapy*	1994	Griswold, L. A. S.

60	*Teachers Survey on Problems with Handwriting: Referral, evaluation, and outcomes	*American Journal of Occupational Therapy*	2004	Hammerschmidt, S. L., & Sudsawad, P.
61	Measuring Knowledge of School-based Occupational Therapy	*American Journal of Occupational Therapy*	2000	Harris, M. B., Alley, S. T.
62	*Pilot of the BOOST-A™: An online transition planning program for adolescents with autism	*Australian Occupational Therapy Journal*	2017	Hatfield, M., Murray, N., Ciccarelli, M., Falkmer, T., & Falkmer, M.
63	*Teacher Perspectives on Collaboration with Occupational Therapists in Inclusive Classrooms: A pilot study	*Journal of Occupational Therapy, Schools, & Early Intervention*	2011	Huang, Y., Peyton, C. G., Hoffman, M., & Pascua, M.
64	Self-regulation Workshop and Occupational Performance Coaching with Teachers: A pilot study	*Canadian Journal of Occupational Therapy*	2016	Hui, C., Snider, L., & Couture, M.
65	*Occupational Therapy in Mainstream Primary Schools: An evaluation of a pilot project	*British Journal of Occupational Therapy*	2009	Hutton, E.
66	Lessons Learned' from Introducing Universal Strategies Designed to Support the Motor and Functional Skills of Reception and Year 1 Children in a Sample of Primary Schools in South East England	*International Journal of Primary, Elementary and Early Years Education*	2009	Hutton, E., Soan, S.
67	*Fine Motor Difficulties: The need for advocating for the role of occupational therapy in schools	*Australian Occupational Therapy Journal*	2007	Jackman, M., & Stagnitti, K.
68	*Effectiveness of an Intensive Handwriting Program for First Grade Students Using the Application LetterSchool: A pilot study	*Journal of Occupational Therapy, Schools, & Early Intervention*	2016	Jordan, G., Michaud, F., & Kaiser, M.
69	Using the LOTCA to Measure Cultural and Sociodemographic Effects on Cognitive Skills in Two Groups of Children	*American Journal of Occupational Therapy*	2011	Josman, N.
70	*The Role of the School-Based Occupational Therapist in Secondary Education Transition Planning: A pilot survey study	*American Journal of Occupational Therapy*	2005	Kardos, M., & White, B. P.
71	*Occupational Therapy in Full-Inclusion Classrooms: A case study from the Moorpark model.	*American Journal of Occupational Therapy*	1996	Kellegrew, D. H., & Allen, D.
72	*Collaborative Consultation: The efficacy of remedial and compensatory interventions in school contexts	*American Journal of Occupational Therapy*	1996	Kemmis, B. L., & Dunn, W.
73	*An Evaluation of Functional, School-based Therapy Services for Children with Special Needs	*Physical and Occupational Therapy in Pediatrics*	1999	King, G. A., McDougall, J., Tucker, M. A., Gritzan, J., Malloy-Miller, T., Alambets, P., et al.

74	*Relationships Between Fine-Motor, Visual-Motor, and Visual Perception Scores and Handwriting Legibility and Speed	*Physical & Occupational Therapy in Pediatrics*	2010	Klein, S., Guiltner, V., Sollereder, P., & Cui, Y.
75	*Use of Online Training Modules for Professional Development with School-based Therapists: Outcome project	*Journal of Occupational Therapy, Schools, & Early Intervention*	2017	Lawdis, K., Baist, H., & C. O. Pittman
76	Social Participation in Schools: A survey of occupational therapy practitioners	*American Journal of Occupational Therapy*	2016	Leigers, K., C., Schneck, C.
77	*Predicting Length of Service Provision in School-Based Occupational Therapy	*Physical & Occupational Therapy in Pediatrics*	2003	Long, D.
78	*Expressão Livre de Jovens por Meio do Fanzine: Recurso para a terapia ocupacional socia [Free Expression Trough Fanzine´s: resource to social occupational therapy]	*Saúde e Sociedade*	2013	Lopes, R. E., Borba, P. L., & Monzeli, G. A.
79	*Work at School: Teacher and parent perceptions about children's participation	*Work*	2010	Lowe, S. & Chapparo, C.
80	*Occupational Therapists' Beliefs and Involvement with Secondary Transition Planning	*Physical & Occupational Therapy in Pediatrics*	2011	Mankey, T. A.
81	*Research on Positive Indicators for Teacher-Child Relationship in Children with Intellectual Disabilities	*Occupational Therapy International*	2015	Matsushima, K., & Kato, T.
82	*Utilization and Satisfaction with Rehabilitation Services in Children with Primary Language Impairment Transitioning to School: Parents' perspective	*International Journal of Disability, Development and Education*	2016	Mazer, B., Dion, K., & Moryoussef, A.
83	*Does Handwriting Instruction Have a Place in the Instructional Day? The relationship between handwriting quality and academic success	*Cogent Education*	2017	Mccarroll, H., & Fletcher, T.
84	*Evaluating a School Skills Programme for Australian Indigenous Children: A pilot study	*Occupational Therapy International*	2006	Mcgarrigle, J., & Nelson, A.
85	Handwriting in 2015: A main occupation for primary school–aged children in the classroom	*Journal of Occupational Therapy, Schools, & Early Intervention*	2016	McMaster, E., Roberts, T.
86	*The Role of Occupational Therapy in Providing Person-centred Transition Services: Implications for school-based practice	*Occupational Therapy International*	2004	Michaels, C. A., & Orentlicher, M. L.
87	*Establishing a Case for Occupational Therapy in Meeting the Needs of Children with Grief Issues in School-Based Settings	*Occupational Therapy in Mental Health*	2007	Milliken, B. E., Goodman, G., Bazyk, S., & Flinn, S.

88	*Examination of the Perceived Efficacy and Goal Setting System (PEGS) With Children with Disabilities, Their Parents, and Teachers	*American Journal of Occupational Therapy*	2006	Missiuna, C., Pollock, N., Law, M., Walter, S., & Cavey, N.
89	*Use of the Medical Research Council Framework to Develop a Complex Intervention in Pediatric Occupational Therapy: Assessing feasibility	*Research in Developmental Disabilities,*	2012	Missiuna, C. A. et al.
90	*Attitudes of Entry Level Occupational Therapy Doctoral Students Towards Inclusion for Students with Disabilities	*Australian Occupational Therapy Journal*,	2007	Mu, K., Franck, L. G., & Konz, C.
91	*Occupational Therapy Students Attitudes Towards Inclusion Education in Australia, United Kingdom, United States and Taiwan	*Occupational Therapy International*	2010	Mu, K., Brown, T., Peyton, C. G., Rodger, S., Huang, Y., Wu, C., Hong, C. S.
92	*Descriptive Study of Occupational Therapists Participation in Early Childhood Transitions	*American Journal of Occupational Therapy*	2008	Myers, C. T.
93	*Factors Associated with Therapists' Involvement in Children's Transition to Preschool	*American Journal of Occupational Therapy*	2011	Myers, C.T., Schneck, C. M., Effgen, S. K., McCormick, K. M., Shasby, S. B.
94	*Making a Difference: Occupational therapy in the public schools	*The Occupational Therapy Journal of Research*	1991	Niehues, A. N., Bundy, A. C., Mattingly, C. F., & Lawlor, M. C.
95	*A Sensorimotor Program for Improving Writing Readiness Skills in Elementary-Age Children	*American Journal of Occupational Therapy*	1990	Oliver, C. E.
96	*The Impact of the Classroom Environment on Defining Function in School-Based Practice	*American Journal of Occupational Therapy*	1997	Orr, C., & Schkade, J.
97	*Effect of an Occupational Intervention on Printing in Children with Economic Disadvantages	*American Journal of Occupational Therapy*	2003	Peterson, C. Q. & Nelson, D. L.
98	Effectiveness of Disc 'O' sit Cushions on Attention to Task in Second-grade Students with Attention Difficulties	*American Journal of Occupational Therapy*	2008	Pfeiffer, B. A., et al.
99	*Content for Educational Programs in School-Based Occupational Therapy from a Practice Perspective	*American Journal of Occupational Therapy*	1994	Powell, N. J.
100	Supporting Occupational Therapists Implementing a Capacity-building Model in Schools	*Canadian Journal of Occupational Therapy*	2017	Pollock, N. A. et al.
101	*Experiences and Perceived Roles of Occupational Therapists Working with Children with Special Learning Needs During Transition to School: A pilot study	*Australian Occupational Therapy Journal*	2002	Prigg, A.
102	*A Short-Term Graphomotor Program for Improving Writing Readiness Skills of First-Grade Students	*American Journal of Occupational Therapy*	2007	Ratzon, N. Z., Efraim, D., & Bart, O.

103	Occupational, Speech and Emotional Therapy with Students Attending Booster Classes	*International Journal of Adolescent Medicine and Health*	2011	Regev, D., & Reiter, S. L.
104	*Investigating the Experiences in a School-based Occupational Therapy Program to Inform Community-based Pediatric Occupational Therapy Practice	*Australian Occupational Therapy Journal*	2014	Rens, L., & Joosten, A.
105	*The School as Social Context: Social interaction patterns of children with physical disabilities	*American Journal of Occupational Therapy*	2002	Richardson, P. K.
106	*Development, Reliability, and Validity of the Handwriting Proficiency Screening Questionnaire (HPSQ)	*American Journal of Occupational Therapy*	2008	Rosenblum, S.
107	*Enhancing Classroom Participation of Students with Intellectual and Developmental Disabilities	*Canadian Journal of Occupational Therapy*	2016	Selanikyo, E., Yalon-Chamovitz, S. & Weintraub, N.
108	*A Systems Approach to Understanding Occupational Therapy Service Negotiations in a Preschool Setting	*Physical & Occupational Therapy in Pediatrics*	2011	Silverman, F., Kramer, P., & Ravitch, S.
109	*Occupational Therapy in Transitioning Adolescents to Post-Secondary Activities	*American Journal of Occupational Therapy*	2003	Spencer, J. E., Emery, L. J., & Schneck, C. M.
110	School-based Practice Patterns: A survey of occupational therapists in Colorado	*American Journal of Occupational Therapy*	2006	Spencer, K. C. et al.
111	*The Role of Inter-professional Collaboration in Developing Inclusive Education: Experiences of teachers and occupational therapists in Slovenia	*International Journal of Inclusive Education*	2017	Suc, L., Bukovec, B., & Karpljuk, D.
112	*Use of a Journal Club for Professional Development: Outcomes in a school-based occupational therapy practice	*Journal of Occupational Therapy, Schools, & Early Intervention*	2016	Szucs, K. A., Benson, J. D., & Corturillo, A.
113	*Effectiveness of Occupational Therapy Strategies for Teaching Handwriting Skills to Kindergarten Children	*Journal of Occupational Therapy, Schools, & Early Intervention*	2011	Taras, H., Brennan, J., Gilbert, A., & Reed, H. E.
114	*The Influence of Ergonomic Factors and Perceptual-Motor Abilities on Handwriting Performance	*American Journal of Occupational Therapy*	1993	Tseng, M. H., & Cermak, S. A.
115	*Enabling Outcomes for Students with Developmental Disabilities through Collaborative Consultation	*The Qualitative Report*	2012	Villeneuve, M. A., & Hutchinson, N. L.
116	*Learning Together for Effective Collaboration in School-based Occupational Therapy Practice	*Canadian Journal of Occupational Therapy*	2012	Villeneuve, M. A., & Shulha, L. M.
117	South Australian School Teachers' Perceptions of Occupational Therapy Report	*Australian Occupational Therapy Journal*	2008).	Vincent, R., Stewart, H., & Harrison, J.

118	The Development of a Culturally Appropriate School-based Intervention for Australian Aboriginal Children Living in Remote Communities: A formative evaluation of the Alert Program® intervention	*Australian Occupational Therapy Journal*	2017	Wagner, B. et al.
119	*Evaluation of Occupational Therapy School-Based Consultation Service for Students with Fine Motor Difficulties	*Canadian Journal of Occupational Therapy*	2006	Wehrmann, S., Chiu, T., Reid, D., & Sinclair, G.
120	*Social Competence and Learning Difficulties: Teacher perceptions	*Australian Occupational Therapy Journal*	2008	Wight, M. & Chapparo, C.
121	*Perceptions and Experiences of Occupational Therapists in Rural Schools	*American Journal of Occupational Therapy*	1996	Wills, K. & Case-Smith, J.
122	*Multisensory Approach to Handwriting Remediation: Perceptions of school-based occupational therapists	*American Journal of Occupational Therapy,*	2002	Woodward, S., & Swinth, Y.
123	*Factors Associated with Comfort Level of School-Based Occupational Therapists in Providing Low-Vision Services	*Occupational Therapy in Health Care*	2015	Workman, M., Vogtle, L. K., & Yuen, H. K.
124	*Languaje, Discriminación y Discapacidad en el Contexto Educativo de Magallanes: Un enfoque de derechos humanos desde la terapia ocupacional [Language, Discrimination and Disability in the School Setting of Magellan Education: A human rights-based approach from occupational therapy]	*Magallania (Punta Arenas)*	2016	Yupanqui, C. A., González, B. M. Á., Llancalahuén, V. Miguel., Quilodrán, O. W., & Toledo, A. C.
125	*A First-Person Exploration of the Experience of Academic Reintegration after First Episode Psychosis	*International Journal of Psychosocial Rehabilitation*	2011	Zafran, H., Tallant, B., & Gelinas, I.
126	*Writing Speed and Legibility of 7-14-Year-old School Students Using Modern Cursive Script	*Australian Occupational Therapy Journal*	1998	Ziviani, J. & Watson-Will, A.
127	*Cognitive versus Multisensory Approaches to Handwriting Intervention: A randomized controlled trial	*OTJR: Occupation, Participation and Health*	2009	Zwicker, J. G., & Hadwin, A. F.

* Articles cited along the manuscript.
